# Supplementary material for: Proteotranscriptomic Profiling of the Toxic Mucus of Kulikovia alborostrata (Pilidiophora, Nemertea)
Source: Toxins (Basel). 2024 Dec 26;17(1):5. doi: 10.3390/toxins17010005 (PMC11768458; doi:10.3390/toxins17010005)
Supplement: Supplementary file 1 [file toxins-17-00005-s001.zip › Supplementary File S4.zip!/ORF 001200 LINALB/1362d454f90accc2/ss_report.pdf]

Email	vas9i-kz@mail.ru
Description	ORF_001200_LINALB
Date	Fri Apr 7 03:17:43 BST 2023
Unique Job ID	1362d454f90accc2

Phyre2

Secondary structure and disorder prediction

	1 . . . . 10 . . . . 20 . . . . 30 . . . . 40 . . . .
Sequence	EFLKTQKRCFSRGEGCAFGGTCCYGSMCSWFKCIRNPKQPIYYETTYTN
Secondary structure	
SS confidence	
Disorder	???? ? ? ? ? ? ? ? ?????? ? ???? ? ? ? ?? ? ? ? ? ? ? ? ? ? ??????? ? ? ? ? ? ??
Disorder confidence	

Confidence Key

High(9) Low (0)

? Disordered (20%)

Alpha helix (37%)

Beta strand (24%)
